# Inhibition of cathepsin B activity attenuates extracellular matrix degradation and inflammatory breast cancer invasion

**DOI:** 10.1186/bcr3058

**Published:** 2011-11-17

**Authors:** Bernadette C Victor, Arulselvi Anbalagan, Mona M Mohamed, Bonnie F Sloane, Dora Cavallo-Medved

**Affiliations:** 1 Department of Pharmacology and Wayne State University, Detroit, Michigan 48201, USA; 2 Department of Zoology, Faculty of Science, Cairo University, Giza, 12613 Egypt; 3 Barbara Ann Karmanos Cancer Institute, Wayne State University, Detroit, Michigan 48201, USA; 4 Department of Biological Sciences, University of Windsor, ON, N9B 3P4 Canada

## Abstract

**Introduction:**

Inflammatory breast cancer (IBC) is an aggressive, metastatic and highly angiogenic form of locally advanced breast cancer with a relatively poor three-year survival rate. Breast cancer invasion has been linked to proteolytic activity at the tumor cell surface. Here we explored a role for active cathepsin B on the cell surface in the invasiveness of IBC.

**Methods:**

We examined expression of the cysteine protease cathepsin B and the serine protease urokinase plasminogen activator (uPA), its receptor uPAR and caveolin-1 in two IBC cell lines: SUM149 and SUM190. We utilized a live cell proteolysis assay to localize in real time the degradation of type IV collagen by IBC cells. IBC patient biopsies were examined for expression of cathepsin B and caveolin-1.

**Results:**

Both cell lines expressed comparable levels of cathepsin B and uPA. In contrast, levels of caveolin-1 and uPAR were greater in SUM149 cells. We observed that uPA, uPAR and enzymatically active cathepsin B were colocalized in caveolae fractions isolated from SUM149 cells. Using a live-cell proteolysis assay, we demonstrated that both IBC cell lines degrade type IV collagen. The SUM149 cells exhibit predominantly pericellular proteolysis, consistent with localization of proteolytic pathway constitutents to caveolar membrane microdomains. A functional role for cathepsin B was confirmed by the ability of CA074, a cell impermeable and highly selective cathepsin B inhibitor, to significantly reduce pericellular proteolysis and invasion by SUM149 cells. A statistically significant co-expression of cathepsin B and caveolin-1 was found in IBC patient biopsies, thus validating our *in vitro *data.

**Conclusion:**

Our study is the first to show that the proteolytic activity of cathepsin B and its co-expression with caveolin-1 contributes to the aggressiveness of IBC.

## Introduction

Inflammatory breast cancer (IBC) is the most lethal type of breast cancer with a three-year survival rate of 42% as compared with 85% for non-IBC [1]. In 2010, an international panel of leading experts recommended the clinical consensus for a minimal standard diagnosis of IBC include erythema occupying at least one-third of the breast, hardening and retraction of the nipple, dimpling (peau d'orange) of the skin, and no response to antibiotic treatment [2]. These clinical characteristics are accompanied by extensive dermal lymphovascular invasion in which tumor emboli are present within dermal lymphatics [3].

Proteases such as the cysteine protease cathepsin B have been implicated in the initiation, promotion and dissemination of cancers including IBC [4-6]. In IBC, high levels of cathepsin B are found to correlate with increases in numbers of metastatic lymph nodes [7]. In tumor cells, cathepsin B redistributes into exocytic vesicles at the cell periphery leading to its secretion and association with the tumor cell surface by binding to the light chain of the annexin II heterotetramer [8,9]. More specifically, we have shown that in colon cancer cells cathepsin B localizes in caveolae [10], a membrane microdomain in which the annexin II heterotetramer is also localized [11]. Downregulation of caveolin-1, the structural protein of caveolae, reduces the cell surface association of cathepsin B and decreases degradation of type IV collagen and invasion by the colon cancer cells, consistent with a functional role for caveolae-associated cathepsin B in invasion [12].

Caveolin-1 was initially hypothesized to be a tumor suppressor in breast cancer [13]. More recent data suggest that high expression of caveolin-1 is a characteristic of triple-negative and other basal-like breast cancers [14], including IBC of a basal phenotype. Indeed, caveolin-1 is highly expressed in both IBC cells and tissues [7,15,16]. We previously hypothesized that the high levels of caveolin-1 expression in bladder, colon, esophageal and prostate cancers promote cell surface proteolytic events that lead to extracellular matrix (ECM) degradation and tumor invasion [17]. For example, proteases of the plasminogen cascade, specifically pro-urokinase plasminogen activator (pro-uPA) and its receptor uPAR have been localized to caveolae [12,18]. These findings may be of functional significance as cathepsin B is capable of processing the zymogen pro-uPA to its active derivative uPA [19] and is found upstream of plasminogen in a proteolytic pathway on the surface of a number of cell lines [20-22]. Moreover, uPAR complexes with caveolin-1 via β1-integrin, an association that has been shown to mediate uPAR-dependent adhesion and β1-integrin-induced signal transduction [23,24]. This suggests that caveolae may serve as sites on the cell surface linking proteolytic and signaling pathways that are involved in tumor invasion.

We hypothesize that participation of cathepsin B in IBC invasion is facilitated by its colocalization at the cell surface with members of the plasminogen cascade and the expression of caveolin-1. Here we demonstrate that cathepsin B as well as uPA and uPAR are associated with caveolar fractions in IBC cells and that cathepsin B is active within these fractions. We confirmed that cathepsin B and caveolin-1 are coexpressed in tissues from IBC patients.

## Materials and methods

### Cell lines

SUM149 and SUM190 human IBC cell lines [25] (a kind gift from Dr. Stephen Ethier, Wayne State University, Detroit, MI, USA) were cultured in Hams F-12 media (Mediatech, Manassas, VA, USA) containing 5 μg/ml insulin, 1 μg/ml hydrocortisone, antibiotics (penicillin/streptomycin), and 5% (SUM149) and 2% (SUM190) fetal bovine serum (HyClone, Logan, UT, USA). Media for SUM190 cells were further supplemented with 5 mM ethanolamine, 10 mM HEPES, 5 μg/ml transferrin, 6.6 ng/ml 3,3',5-triiodo-L-thyronine sodium salt, 8.7 ng/ml sodium selenite, and 1 mg/ml bovine serum albumin. All cells were maintained in 5% CO_2_/humidified atmosphere at 37°C. Unless otherwise stated all tissue culture reagents were from Sigma Aldrich (St. Louis, MO, USA).

### Preparation of cell lysates and conditioned media

IBC cells were grown on plastic to 70% confluency and then serum-starved overnight. Conditioned media were collected and centrifuged at 100 × *g *at 4°C to remove whole cells, and then re-centrifuged at 800 × *g *at 4°C to remove cell debris. All conditioned media samples were concentrated to equal volumes in Ultrafree-0.5 PBGC Centrifugal Filter Units with 5 kDa molecular weight cut off Biomax Membranes (Millipore, Billerica, MA, USA). Cells were harvested in lysis buffer (10 mM Tris, pH 7.5, 150 mM NaCl, 1% Triton X-100, 60 mM octylglucoside) and then passed 10 times through a syringe with a 20-gauge needle and centrifuged for five minutes at 10,000 × *g *at 4°C. Supernatants were recovered and protein concentrations were quantified using micro-BCA reagents according to the manufacturer's instructions (Pierce, Rockford, IL, USA).

### Preparation of 3D reconstituted basement membrane overlay cultures

Sixty mm dishes were coated with 300 μl of reconstituted basement membrane (rBM; Cultrex, Trevigen, Gaithersburg, MD, USA) and allowed to solidify for 15 minutes at 37°C. Cells (1 × 10^6^) were seeded on top of solidified rBM and grown in complete media containing 2% rBM. Within 24 hours, cells formed 3D spheroid structures.

### Isolation of caveolae-enriched fractions

Non-detergent and detergent-based protocols were used to prepare caveolae-enriched membrane fractions of IBC cells [26]. In the non-detergent based method, cells grown as a two-dimensional (2D) monolayer on plastic (4 × 100 mm dishes) and three-dimensional (3D) on rBM (4 × 60 mm dish) for two and five days, respectively, were washed with PBS, collected into 500 mM sodium carbonate buffer, pH 11.0, homogenized in a Dounce homogenizer on ice and sonicated three times for 10 seconds each. A discontinuous sucrose gradient was prepared as previously detailed [26]. Briefly, the cell homogenate was mixed thoroughly with an equal volume of 90% (w/v) sucrose, then overlaid with 35% (w/v) sucrose and 5% (w/v) sucrose and subsequently ultracentrifuged (185,000 × *g*) for 19 hours at 4°C. Fractions of 1 mL were collected and equal-volume aliquots of fractions 3-11 were analyzed by SDS-PAGE and immunoblotting.

A successive detergent-based method of cell fractionation was also employed to separate Triton X-100-soluble (TS) and Triton X-100-insoluble (TI) membrane fractions [27]. 3D cultures were prepared and grown, as described above, for two days and thereafter washed with cold PBS, incubated with 300 μl of lysis buffer containing 1% Triton X-100 for 20 minutes on ice, collected and centrifuged at 14,000 × *g *for 10 minutes at 4°C. The supernatant (TS fraction) was collected and the pellet was resuspended in 300 μl of lysis buffer containing 1% Triton X-100 plus 60 mM octylglucoside, incubated on ice for 20 minutes, passed through a syringe with a 21.5-gauge needle and centrifuged at 14,000 × *g *for 10 minutes at 4°C. The supernatant (TI fraction) was recovered. Equal-volume aliquots of each fraction were analyzed by SDS-PAGE and immunoblotting.

### SDS-PAGE and immunoblotting

Cell lysates and conditioned media samples were equally loaded on the basis of the protein concentration of the respective cell lysates, separated by SDS-PAGE (10 or 12%) under either reducing or non-reducing conditions, transferred to nitrocellulose membranes and immunoblotted with primary antibodies against human cathepsin B (1:4000; [28]), β1-integrin (1:3000; a kind gift from Dr. Kenneth Yamada, National Institute of Dental and Craniofacial Research, National Institutes of Health, Bethesda, MD, USA), uPA (1:2000; Abcam, Cambridge, MA, USA), uPAR (1:2000; Abcam, Cambridge, MA, USA), caveolin-1 (1:4000; BD Biosciences, Bedford, MA, USA), or β-tubulin (1:1500; prepared from hybridoma E7 cells, Developmental Studies Hybridoma Bank, National Institute of Child Health and Human Development, University of Iowa, Iowa City, IA, USA) and secondary antibodies conjugated with horseradish peroxidase (HRP; 1:10,000; Pierce) in TBS buffer (20 mM Tris, pH 7.5, 0.5 M NaCl) containing 0.5% Tween 20 and 5% (w/v) non-fat dry milk. After washing, bound antibodies were detected by enhanced chemiluminescence according to the manufacturer's instructions (PerkinElmer, Waltham, MA, USA).

### Cathepsin B activity assay

Cathepsin B activity was measured using the synthetic fluorometric substrate Z-Arg-Arg-NHMec as previously described [10]. Briefly, equal-volume aliquots of TS and TI fractions were incubated with activator buffer for 15 minutes at 37°C. Following this activation step, 150 μM Z-Arg-Arg-NHMec (pH 6.0) was added to the assay buffer and fluorescence was measured in triplicate, at one-minute intervals for 30 minutes, at an excitation of 360 nm and an emission of 465 nm. Data are represented as relative fluorescent units and presented as mean ± standard deviation (SD) of three independent experiments. Statistical significance was determined by a two-tailed t-test with assumed equal variance.

### Live-cell proteolysis assay

Proteolytic cleavage of DQ-collagen IV substrate (Invitrogen, Carlsbad, CA, USA) by live IBC cells was imaged in real time and quantified as previously described [29,30]. Briefly, IBC cells (2.5 × 10^4^) were seeded on round glass coverslips coated with rBM containing 25 μg/ml DQ-collagen IV substrate and incubated at 37°C for 40 minutes to allow for cell attachment before adding complete media containing 2% rBM. Following 24 to 48 hours of incubation at 37°C and 5% CO_2_, live cells were imaged (37°C and 5% CO_2_) with a Zeiss LSM 510 META NLO confocal microscope using a 40× Plan neofluar (N.A., 0.7) objective. DQ-collagen IV cleavage products were observed as green fluorescence. Where specified, the assay was performed in the presence of 10 μM CA074 (Peptides International, Louisville, KY, USA). Statistical significance was determined by a two-tailed t-test with assumed equal variance.

### Invasion assay

Cell culture inserts (8.0 μm transparent PET membranes (BD Biosciences, Franklin Lakes, NJ, USA)), were coated with 2 mg/ml rBM and incubated in a 24-well plate at room temperature to permit rBM solidification. SUM149 cells (5.0 × 10^4^) in serum-free media were seeded onto the rBM-coated inserts and incubated for 24 hours at 37°C in the presence of dimethyl sulfoxide (DMSO, vehicle control) or 10 μM CA074. The stimulant for invasion was complete media. Cells that had not invaded were removed with a cotton swab. Cells that had invaded were fixed with 3.7% formaldehyde (Polysciences, Inc., Warrington, PA, USA), stained with 4',6-diamidino-2-phenylindole (Invitrogen, Carlsbad, CA, USA) and imaged at 20× magnification with a Zeiss Axiophot conventional epifluorescent microscope. Ten random microscopic fields per filter were analyzed. The number of cells that invaded was assessed by counting nuclei with MetaMorph™ image analysis software (Molecular Devices, Sunnyvile, CA, USA). Statistical significance was determined by a two-tailed t-test with assumed equal variance.

### Immunocytochemical staining

Intracellular co-staining was performed at room temperature on 2D (permeabilized with 0.1% saponin) and 3D cultures (permeabilized with 0.2% Triton X-100). The cultures were incubated with the following primary antibodies diluted in 0.2% Triton X-100/PBS: rabbit anti-caveolin-1 (1:50), mouse anti-uPA (1:25), and mouse anti-uPAR (1:12.5). Secondary antibodies (donkey anti-rabbit Alexa Fluor 488 and donkey anti-mouse Alexa Fluor 555 (Invitrogen, Carlsbad, CA, USA)) were diluted (1:5000) in 0.2% Triton X-100/PBS plus 5% normal donkey serum. All immunocytochemical staining was imaged on a Zeiss LSM 510 META NLO confocal microscope using either a 100× oil Plan neofluar (N.A., 1.3) objective (2D cultures) or 40X Plan apofluar (N.A., 0.7) objective (3D cultures).

### Immunohistochemical staining of patient samples

Institutional review board approval from Ain Shams University ethics committee and the National Cancer Institute, Cairo University, along with patient consent forms, were obtained for the purpose of patient enrollment in this study. Inclusion criteria for patients and collection of tissue samples were as previously described [7]. Pre-treated formalin fixed paraffin embedded IBC (*n *= 23) and non-IBC (*n *= 27) patient tissue samples were subjected to immunohistochemical (IHC) analysis [7]. Briefly, tissue sections were incubated for one hour at room temperature with either monoclonal anti-caveolin-1 (1:150) or polyclonal anti-cathepsin B (1:500) primary antibodies. A second incubation was performed with HRP rabbit/mouse (EnVision + Dual Link System-HRP (DAB +)) for 45 minutes. Nuclei were counterstained with hematoxylin, sections were mounted with Permount^® ^and imaged. *P *values for the relation between cathepsin B expressing breast carcinoma cells, of both IBC and non-IBC, and caveolin-1 protein expression were assessed with a chi-square test.

## Results

### Cathepsin B and uPA expression and secretion are similar in SUM149 and SUM190 cells

**Figure 1:**
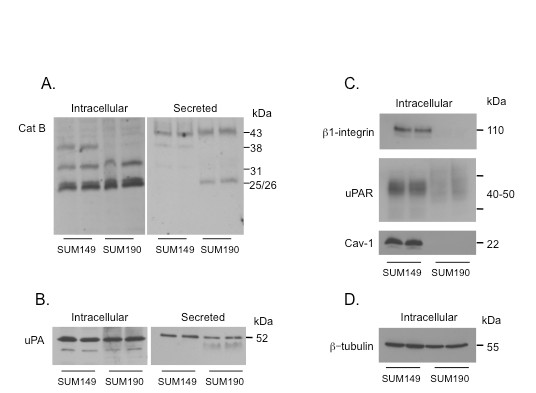
**Profile of cathepsin B, uPA, uPAR, β1-integrin and caveolin-1 expression in SUM149 and SUM190 cells**. Expression of intracellular proteins was analyzed in duplicate samples of cell lysates, whereas expression of secreted proteins was analyzed in duplicate samples of conditioned media. Lysates and conditioned media of 2D cultures of SUM149 and SUM190 cells were equally loaded based on the protein concentration of the respective cell lysate and resolved by 10% SDS-PAGE. Proteins were transferred to a nitrocellulose membrane and immunoblotted with **(a) **an anti-cathepsin B (cat B) polyclonal antibody (bands represent proform (43 kDa), intermediate (38 kDa), single chain mature (31 kDa), and heavy chain (25/26 kDa) of double chain mature cathepsin B); **(b) **an anti-uPA antibody; **(c) **anti-uPAR, anti-β1-integrin, anti-caveolin-1 (cav-1) antibodies and **(d) **anti-β-tubulin monoclonal antibody with the concentration of α-tubulin in the cell lysate serving as a loading control for both cell lysates and their respective conditioned media. uPA, urokinase-type plasminogen activator; uPAR, urokinase receptor.

Cathepsin B has been linked to both pericellular and intracellular proteolysis in breast cancer cells [31]. Here we examined two IBC cell lines, SUM149 and SUM190, and found that the two cell lines express comparable levels in the cell lysates of the mature active forms of cathepsin B (single chain (31 kDa) and double chain (25/26 kDa heavy chain + 5 kDa light chain)) and secrete comparable levels of the inactive precursor form, procathepsin B (43 kDa) (Figure 1a). A 38 kDa intermediate form of the enzyme was present only in lysates of the SUM149 cells and mature double chain cathepsin B was secreted only from SUM190 cells (Figure 1a); these differences between the two cell lines are likely to be due to variations in the extent of processing of the proenzyme into the various mature forms, as we have observed in a variety of human tumor tissues [28]. We also compared the expression of uPA in both cell lines because cathepsin B activates both soluble and cell surface bound pro-uPA [19-22]. Both IBC cell lines expressed similar levels of intracellular pro-uPA; however, secretion of pro-uPA was slightly higher from the SUM149 cell line (Figure 1b).

### uPAR, caveolin-1 and β1-integrin expression is higher in SUM149 than in SUM190 cells

The cell surface receptor for uPA is uPAR [32,33], a GPI-anchored membrane glycoprotein that has been localized to caveolae in a wide variety of cells [12,23,24]. We found that SUM149 cells expressed more receptor than did SUM190 cells (Figure 1c). Caveolin-1 and β1-integrin form a trimeric complex with uPAR within caveolae [24], a complex that mediates cell adhesion and migration. Therefore, we also compared expression of caveolin-1 and β1-integrin in the two cell lines. There was a striking dissimilarity in levels of the two proteins (Figure 1c), paralleling the difference in expression of uPAR. The differences observed in expression of cav-1, uPAR, and β1-integrin, which are involved in localization and activation of uPA and cathepsin B at the cell surface, suggest that cell surface or pericellular proteolysis may also differ between the two cell lines.

### SUM149 and SUM190 cells exhibit different patterns of proteolysis of type IV collagen

**Figure 2:**
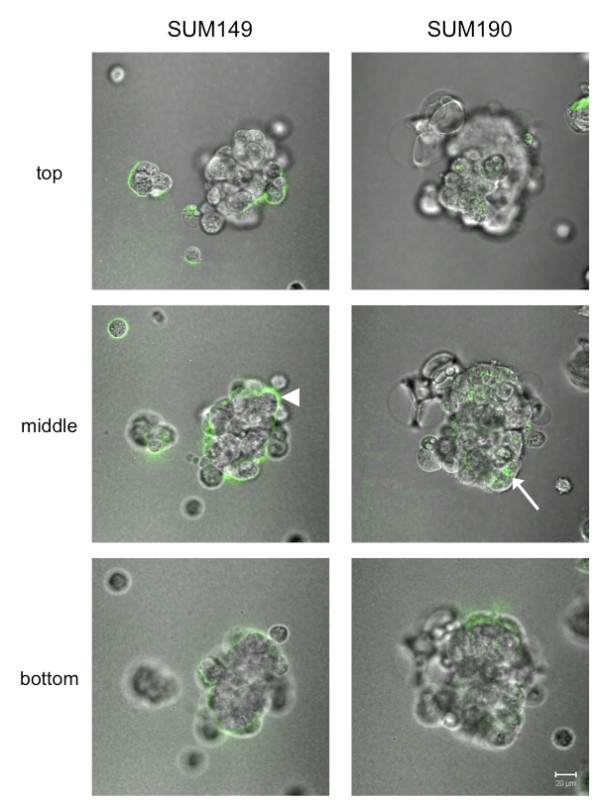
**Live-cell proteolysis assay revealed disparate proteolytic patterns in 3D cultures of SUM149 and SUM190 cells**. SUM149 and SUM190 cells were seeded onto glass-coverslips coated with reconstituted basement membrane containing DQ-collagen IV. Following overnight incubation at 37°C, cells and DQ-collagen IV cleavage products (green) were imaged using confocal microscopy (see Materials and Methods). 3D structures of live SUM149 and SUM190 cells degraded DQ-collagen IV pericellularly (arrowhead) and predominantly intracellularly (arrow), respectively. Confocal Z stack images were reconstructed in 3D [see Additional files 1 and 2] utilizing Volocity™ software (PerkinElmer, Waltham, MA, USA). Representative merged images of DIC and green channels of the equatorial plane (middle) and planes equidistant, 20 μm, from the equatorial plane (top and bottom) are also shown. Bar, 20 μm.

We used a functional live-cell proteolysis assay to assess in real-time degradation of DQ-collagen IV by SUM149 and SUM190 cells. DQ-collagen IV cleavage products (green fluorescence) were present in cultures of both cell lines; however, there were distinct differences in localization of cleavage products (Figure 2) [see Additional files 1 and 2]. Cleavage products were predominantly pericellular in the SUM149 cell line indicating degradation by extracellular proteases at the cell surface. In contrast, in the SUM190 cell line a distinct punctate fluorescence pattern was present intracellularly, suggesting that the cleavage products were in intracellular vesicles. This may reflect proteolysis either intracellularly in the vesicles or extracellularly with degradation fragments taken up into the vesicles by endocytosis.

### uPA, uPAR and β1-integrin colocalize in caveolae-enriched fractions of SUM149 cells

**Figure 3:**
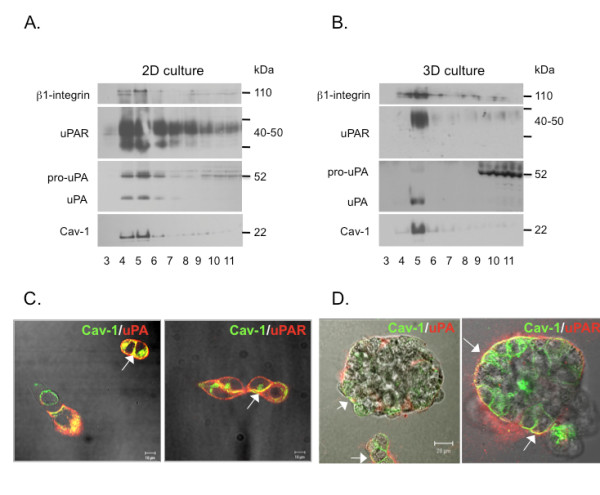
**Localization of uPA, uPAR and β1-integrin to caveolae-enriched fractions of SUM149 cells**. Isolation of caveolae from SUM149 cells grown in **(a) **2D or in **(b) **3D culture was performed using a non-detergent method as described in Materials and Methods. Equal-volume aliquots of fractions 3-11 were analyzed by SDS-PAGE and immunoblotted with antibodies against uPA, uPAR, β1-integrin, or caveolin-1. Merged DIC and fluorescent images are representative images of SUM149 **(c) **2D or **(d) **3D cultures immunostained for caveolin-1 (green) and uPA (red) or uPAR (red). Colocalization of the two proteins appears yellow (arrows). Confocal microscopy was performed as described in Materials and Methods. Bar, 10 μm for 2D cultures; 20 μm for 3D cultures. uPA, urokinase-type plasminogen activator; uPAR, urokinase receptor.

As levels of expression of caveolin-1, uPAR, and β1-integrin were high in SUM149 cells and we have previously shown that uPA, uPAR, and β1-integrin colocalize in caveolar fractions of colorectal carcinoma cells [12], we isolated caveolae-enriched fractions from SUM149 cells. We used a non-detergent-based method that employs a sodium carbonate buffer for cell lysis followed by subcellular fractionation in a sucrose gradient [26]. Analysis of caveolae-enriched fractions (i.e., fractions in which caveolin-1 was primarily distributed) revealed that uPA, its receptor uPAR and β1-integrin were all present in these fractions (Figures 3a and 3b). This was observed for SUM149 cells grown either as 2D or 3D cultures. For cell fractionation studies, sufficient starting material is more readily obtained from 2D cultures. We prefer, however, to analyze 3D cultures because they more closely resemble the *in vivo *phenotype [34] and have been shown to be predictive of drug responses *in vivo *[35]. In agreement with our fractionation studies, immunostaining of 2D (Figure 3c) and 3D (Figure 3d) cultures of SUM149 cells showed that uPA and uPAR colocalized with caveolin-1, consistent with a role for caveolin-1 in the distribution of these proteins to the cell surface.

### Active cathepsin B localizes to caveolae of SUM149 cells

**Figure 4:**
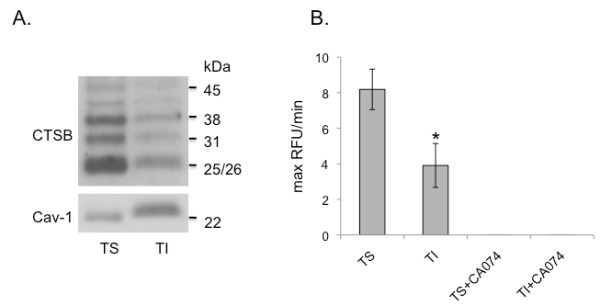
**Active cathepsin B is localized to caveolae-enriched fractions of SUM149 3D cultures**. Caveolae from SUM149 3D cultures were isolated by a detergent-based method, as described in Materials and Methods, that separates Triton X-100-soluble (TS; non-caveolae) from Triton X-100-insoluble (TI; caveolae) cellular fractions. **(a) **Equal-volume aliquots of each fraction were analyzed by SDS-PAGE and immunoblotted with antibodies against cathepsin B or caveolin-1. **(b) **TS and TI fractions were assayed for enzymatic activity against the synthetic cathepsin B substrate Z-Arg-Arg-NHMec. Cathepsin B activity in both TS and TI fractions was inhibited by 10 μM CA074; *, *P *< 0.01.

We have previously shown, using both detergent and non-detergent methods, that cathepsin B localizes to caveolae-enriched fractions of colon carcinoma and endothelial cells [10,12,26]. Cathepsin B activity is destroyed in the non-detergent-based method as the cells are lysed in sodium carbonate buffer at pH 11. Therefore, to assess localization of cathepsin B to caveolae-enriched fractions of SUM149 3D cultures, we utilized a detergent-based method that separates TS from TI membrane components. As caveolae are insoluble in 1% Triton X-100, the TI fraction contains caveolae and associated proteins and the TS fraction contains proteins from cellular components that are solubilized by the detergent. As expected more caveolin-1 was observed in the TI fraction than the TS fraction (Figure 4a). Mature forms of cathepsin B were present in the TI fractions (Figure 4a). Substantially higher levels of mature cathepsin B were present in the TS fractions containing lysosomes, the predominant localization of cathepsin B, which is a lysosomal enzyme. Cathepsin B activity was observed in both caveolar and lysosomal fractions (Figure 4b), at levels comparable with the levels of mature cathepsin B detected by immunoblotting (Figure 4a). The enzymatic activity in the isolated caveolar and lysosomal fractions was completely abrogated by CA074, a highly selective inhibitor for cathepsin B [36]. The presence of cathepsin B activity in the TI fractions would be consistent with a functional role for cathepsin B at the cell surface.

### Inhibition of cathepsin B reduces type IV collagen degradation and invasion by IBC cells

**Figure 5:**
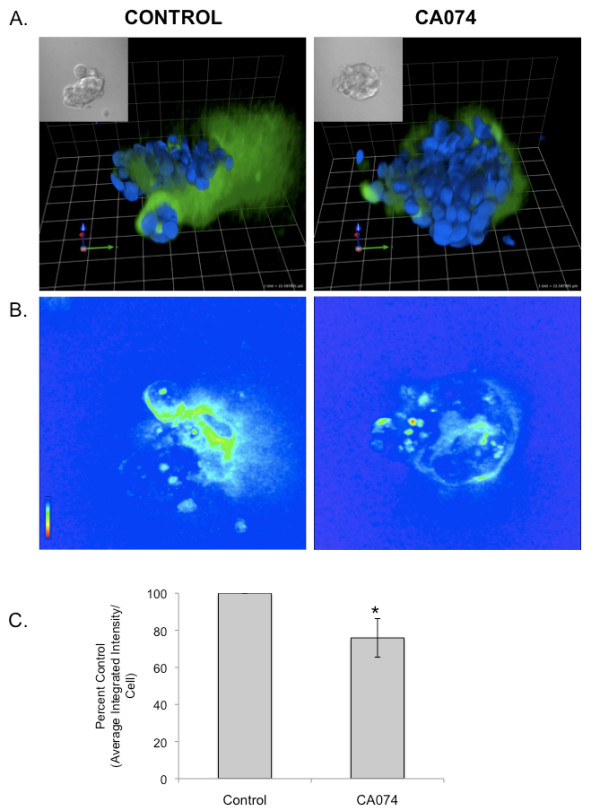
**Inhibition of cathepsin B activity reduces degradation of DQ-collagen IV by SUM149 3D cultures**. SUM149 cells were grown as 3D reconstituted basement membrane overlay cultures containing DQ-collagen IV in the presence of either DMSO (vehicle control) or 10 μM CA074. Confocal Z stack images were captured and used to generate 3D reconstructions (see legend for Figure 2 for detail) [see Additional files 3 and 4]. **(a) **Representative 3D reconstructions of DQ-collagen IV degradation products (green), SUM149 nuclei (stained with Hoechst 33342, blue) and corresponding DIC images (insets). **(b) **Corresponding intensity map (red being most intense and blue least intense) of DQ-collagen IV degradation products. Magnification, 40×. **(c) **Quantification of proteolysis in the entire volume of 3D structures measured as the average integrated intensity of fluorescence per cell and expressed as percent control. Results from three independent experiments are presented as mean ± standard deviation; *, *P *< 0.02.

**Figure 6:**
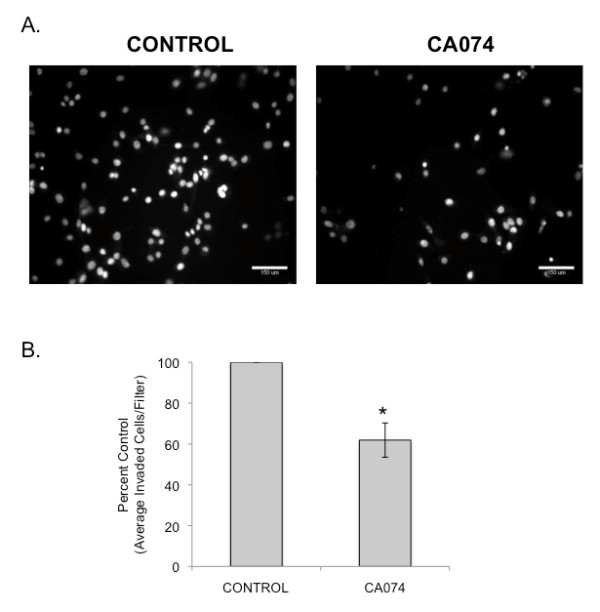
**Inhibition of cathepsin B activity attenuates invasiveness of SUM149 cells**. SUM149 cells were subjected to transwell invasion assays in the presence of DMSO (vehicle control) or 10 μM CA074. **(a) **Images are SUM149 cells that have invaded through rBM-coated filters; bar, 150 μm. **(b) **Quantification of invaded cells was performed by counting the average number of nuclei from 10 fields of view per filter (using Metamorph software) and expressed as percent control. Results from three independent experiments are presented as mean ± standard deviation; *, *P *< 0.05.

Our live-cell proteolysis assays indicated that SUM149 cells degrade DQ-collagen IV pericellularly and our fractionation and enzymatic assays indicated that active cathepsin B is associated with caveolae of these cells. Therefore, we examined whether inhibiting cathepsin B would reduce pericellular degradation of DQ-collagen IV. For these cell-based assays, we used CA074, which is cell-impermeant and thus would only reduce cathepsin B activity outside the SUM149 cells. We used our previously established methods to quantify degradation of DQ-collagen IV on a per cell basis throughout the entire volume of SUM149 3D cultures [29]. We demonstrated that CA074 significantly attenuated degradation of DQ-collagen IV (Figure 5) [see Additional files 3 and 4) and invasion (Figure 6), suggesting that cathepsin B contributes to pericellular degradation and invasion by SUM149 cells.

### Cathepsin B and caveolin-1 are co-expressed in IBC tissues *in vivo *

**Figure 7:**
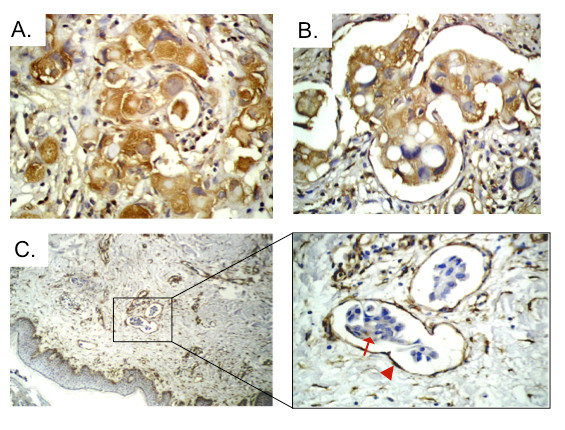
**Immunohistochemical staining for cathepsin B and caveolin-1 in paraffin sections of inflammatory breast cancer tissues**. **(a) **Diffuse cytoplasmic staining for cathepsin B in tumor and stromal cells (magnification, 40×). **(b) **Cathepsin B staining in tumor emboli (magnification, 40×). **(c) **Caveolin-1 staining in tumor emboli (arrow) and dermal lymphatic endothelial cells (arrowhead) (magnification: left panel, 10×; right panel, 40×).

To demonstrate that there is an association between cathepsin B and caveolin-1 *in vivo*, we immunostained paraffin-embedded tissue samples from IBC and non-IBC patients for cathepsin B and caveolin-1. IBC patients in this study ranged in age from 29 to 60 years (mean ± SD = 41 ± 8); non-IBC patients ranged in age from 33 to 67 years (mean ± SD = 50 ± 9). Tumor grade analysis revealed that 65% and 78% of the IBC and non-IBC samples, respectively, were grade I or II and 35% and 22% of the IBC and non-IBC samples, respectively, were grade III. For further clinical and pathological characterization of the IBC and non-IBC samples, see [7]. In the IBC tissues, we observed strong expression of cathepsin B in tumor cells and in tumor emboli within dermal lymphatics and moderate expression in stromal cells (Figures 7a and 7b). There was heterogeneous staining for caveolin-1 in tumor emboli (Figure 7c). Endothelial cells of dermal lymphatics containing tumor emboli that stained strongly for caveolin-1, consistent with the known high levels of caveolae in endothelial cells [37]. Furthermore, there was a difference between IBC and non-IBC carcinoma cells in regard to co-expression of cathepsin B and caveolin-1. Of IBC tumor cells that express cathepsin B (score of ++ or +++), 70% also expressed caveolin-1 (score of ++ or +++), whereas only 19% of non-IBC tumor cells showed this co-expression (*P *= 0.001) [see Additional file 5].

## Discussion

IBC is a rare but highly aggressive form of breast cancer with symptoms that develop rapidly (i.e., weeks or months) after initial diagnosis [38]. Current IBC-specific treatments are very limited and development of new therapeutics is needed, specifically therapies against pathways that mediate the aggressive IBC phenotype. IBC is, however, not amenable to laboratory investigations as there are only two commercially available IBC cell lines (SUM149 and SUM190) for *in vitro *investigations and one human xenograft model (MARY-X) [25,39]. Our approach in this study was to investigate which proteases expressed by SUM149 and SUM190 IBC cells are associated with caveolin-1, which is highly expressed in IBC *in vivo *[7,16], participate in ECM degradation and invasion and confirm the presence of these proteases in IBC patient samples. Our findings implicate cathepsin B as one contributor to the aggressive IBC phenotype.

Cathepsin B had previously been shown to activate pro-uPA, a serine protease and member of the plasminogen cascade involved in ECM degradation, matrix metalloproteinase (MMP) activation, and tumor cell invasion [19]. In SUM149 cells, uPA and its receptor uPAR colocalize with active cathepsin B in caveolae. The presence of active cathepsin B in caveolae of IBC cells suggests a potential role for this enzyme in pericellular proteolysis as was previously shown in colon carcinoma cells. Downregulation of caveolin-1 in the colon carcinoma cells decreases cathepsin B localization to caveolae in parallel with decreases in ECM degradation and cell invasion [12]. In the SUM149 cells in this study, which co-express cathepsin B and caveolin-1, we determined that degradation of type IV collagen was predominantly pericellular and that a cell impermeant cathepsin B inhibitor reduced their degradation of type IV collagen and invasion. Although significant, the lack of complete inhibition suggests that cathepsin B was only one of several proteases in the SUM149 cells that degrade type IV collagen and mediate invasion. Rao and colleagues have demonstrated that downregulation of cathepsin B and MMP9 more effectively reduces invasion of prostate tumor cells *in vitro *and tumor growth *in vivo *than downregulation of either cathepsin B or MMP9 [40]. On the other hand, downregulation of cathepsin B and uPAR more effectively reduces invasion of human glioma cells *in vitro *and *in vivo *in an intracranial xenograft model than downregulation of either cathepsin B or uPAR [41]. The above studies indicate that proteases can compensate for one another. There is in addition functional redundancy, including among cysteine cathepsins [6,42]. A striking example is the redistribution of active cathepsin × to the surface of mammary tumor cells isolated from mice deficient in cathepsin B that had been crossed with mice predisposed to develop mammary cancer, in this case MMTV-PyMT transgenic female mice. Moreover, cathepsin × neutralizing antibodies reduced invasion of the cathepsin B-deficient mammary tumor cells, a result that is consistent with cathepsin × compensating for the absence of cathepsin B [43].

The studies above illustrate that *in vitro *assays such as invasion and ECM degradation assays are meaningful surrogates for *in vivo *tumor endpoints. In a recent collaborative study (N Withana, BF Sloane and BS Parker, unpublished observations), we demonstrated that either knockdown or inhibition of cathepsin B in 4T1 mammary carcinoma cells reduced collagen degradation *in vitro*, as assessed by our live-cell proteolysis assay, and bone metastasis *in vivo*. Here using this live-cell proteolysis assay, we observed differences in the degradation of type IV collagen by the two IBC cell lines. These differences were further supported by variations in expression and secretion of proteases and known caveolae-associated proteins. We propose that these differences may be due, in part, to the differences in receptor status of the two IBC cell lines. Although both lack estrogen and progesterone receptors, only SUM149 is HER2 negative, classifying it as a triple-negative breast cancer cell line. Triple-negative breast cancer is a subtype of breast cancer characterized as extremely aggressive, having a poor prognosis and difficult to treat with high risks of both recurrence and death [44,45]. A role for cathepsin B has previously been reported in several triple-negative breast cancer cell lines that are not IBC (e.g., BT20, BT549, MCF-10AneoT, and MDA-MB-231) [31,46-48], and inhibition of active cathepsin B in these cells reduced their invasion *in vitro*. *In vivo *studies also provide evidence for an association between cathepsin B and breast cancers that are triple negative [49] (N Withana, BF Sloane and BS Parker, unpublished observations). In addition, a transgenic mouse model of mammary cancer characterized by loss of hormone receptors with progression of disease [50] exhibited both reduced primary tumor growth and lung metastases when deficient in cathepsin B [51], suggesting a link between cathepsin B expression and/or activity and invasive and metastatic breast disease. This may be especially true in IBC as expression of cathepsin B was found to be positively correlated with lymph node metastasis in IBC tissues, a correlation not observed in non-IBC tissues [7]. As such, cathepsin B has been proposed to be a prognostic marker for IBC and potentially a component of a proposed molecular signature for IBC that already includes caveolin-1 [16]. Our current findings show that cathepsin B and caveolin-1 were co-expressed in tumor cells of IBC patient samples and not in those of non-IBC patients. Ongoing work will demonstrate if this co-expression is elevated in triple-negative breast cancer patients other than those diagnosed with IBC.

Another enzyme implicated in the aggressive IBC phenotype is RhoC GTPase, which is increased in expression and activity [52,53]. We speculate that there may be a network that links cathepsin B, caveolin-1, and Rho signaling pathways in IBC. In colon, prostate, and non-IBC tumors, phosphorylated caveolin-1 has been shown to promote migration and invasion via a Rho signaling pathway [54]; this has not yet been assessed in IBC. A P132L mutation in caveolin-1 confers a dominant-negative effect on invasiveness of human schirrhous breast cancers [55] and upregulates genes involved in invasiveness and metastasis, including Rho-related signaling molecules and genes expressed by stem cells [56]. Studies in MDA-MB-231 breast carcinoma cells by Bourguignon and colleagues [57] connect Rho, caveolin-1, and cathepsin B. Rho kinase signaling events, mediated upstream by CD44-NHE1 interactions localized to lipid microdomains containing caveolin-1, result in acidification of the microenvironment surrounding breast cancer cells, activate secretion of cathepsin B and promote cellular invasiveness. We have previously shown that slight acidification of the microenvironment of a variety of tumors (melanoma, colon, and breast) increases secretion and activity of cathepsin B and proteolysis of type IV collagen [8,58,59]. Whether there is a universal link between Rho, caveolin-1, cathepsin B, and acidification of the tumor microenvironment has not yet been evaluated.

## Conclusions

Our *in vitro *data demonstrate that the cysteine protease cathepsin B participates in degradation of type IV collagen and invasion by IBC cells, likely through its association with proteolytic pathways in caveolae at the cell surface. These *in vitro *results are supported by the highly significant co-expression of cathepsin B and caveolin-1 in IBC cells of patient samples. Our study is the first to show that cathepsin B, in association with caveolin-1, contributes to the aggressive phenotype of IBC. This is significant as both cathepsin B and caveolin-1 are potential biomarkers for the disease. Therefore, clinical approaches targeting active cathepsin B may prove efficacious in the treatment of patients with IBC. Combinational therapeutic approaches targeting both cathepsin B and uPAR have been shown by Rao and colleagues to inhibit tumor progression [60] and neovascularization in malignant glioma [61]. Several protease targeted drugs are routinely used in the clinic, for example, ACE inhibitors, and others are in pre-clinical development, including neutralizing antibodies against both cathepsin B and uPA for cancer treatment (for review, see [62]).

## Abbreviations

2D: two-dimensional; 3D: three-dimensional; DMSO: dimethyl sulfoxide; ECM: extracellular matrix; HRP: horseradish peroxidase; IBC: inflammatory breast cancer; IHC: immunohistochemical; MMP: matrix metalloproteinase; PBS: phosphate-buffered saline; pro-uPA: pro-urokinase-type plasminogen activator; rBM: reconstituted basement membrane; SD: standard deviation; TI: Triton X-100-insoluble; TS: Triton X-100-soluble; uPAR: urokinase receptor.

## Competing interests

The authors declare that they have no competing interests.

## Authors' contributions

BCV, AA, MMM and DCM carried out the experiments. BCV, MMM, BFS and DCM made substantial contributions to concept and design of experiments as well as drafting and/or revising the manuscript. All authors have read and approved the manuscript.

## Supplementary Material

Additional file 1 **supplementary movie 1**. **Degradation of DQ-collagen IV by SUM149 cells grown in 3D rBM overlay culture**. DQ-collagen IV cleavage products (green) were imaged, and superimposed on DIC images. Z-stack images captured by confocal microscopy were used to generate 3D reconstructions. Click here for file

Additional file 2 **supplementary movie 2. Degradation of DQ-collagen IV by SUM190 cells grown in 3D rBM overlay culture**. DQ-collagen IV cleavage products (green) were imaged, and superimposed on DIC images. Z-stack images captured by confocal microscopy were used to generate 3D reconstructions. Click here for file

Additional file 3 **supplementary movie 3**. **Degradation of DQ-collagen IV by SUM149 cells grown in 3D rBM overlay culture incubated with dimethyl sulfoxide (vehicle control)**. DQ-collagen IV cleavage products (green) and nuclei, stained with DAPI (blue), were imaged. Z-stack images captured by confocal microscopy were used to generate 3D reconstructions. Click here for file

Additional file 4 **supplementary movie 4**. **Degradation of DQ collagen IV by SUM149 cells grown in 3D rBM overlay culture incubated with 10 μM CA074**. DQ-collagen IV cleavage products (green) and nuclei, stained with DAPI (blue), were imaged. Z-stack images captured by confocal microscopy were used to generate 3D reconstructions. Click here for file

Additional file 5 **supplementary table 1. Co-expression of caveolin-1 and cathepsin B in carcinoma cells of IBC versus non-IBC tissues**. Immunohistochemical scores of 0 and + were considered negative and scores of ++ and +++ were considered positive. Data presented as number of patients (%). Chi-square = 11.3 (degrees of freedom = 1). IBC, inflammatory breast cancer. Click here for file
