# Supplementary material for: Inhibition of cathepsin B activity attenuates extracellular matrix degradation and inflammatory breast cancer invasion
Source: Breast Cancer Res. 2011 Nov 17;13(6):R115. doi: 10.1186/bcr3058 (PMC3326557; doi:10.1186/bcr3058)
Supplement: Additional file 5 — supplementary table 1. Co-expression of caveolin-1 and cathepsin B in carcinoma cells of IBC versus non-IBC tissues. Immunohistochemical scores of 0 and + were considered negative and scores of ++ and +++ were considered positive. Data presented as number of patients (%). Chi-square = 11.3 (degrees of freedom = 1). IBC, inflammatory breast cancer. [file bcr3058-S5.PDF]

	**Caveolin-1**		**total**
	negative	positive	
**IBC**	7 (30.4)	16 (69.6)	23
**non-IBC**	17 (81.0)	4 (19.0)	21
**total**	24	20	44
	P value = 0.001		
